# Orbital angular momentum of single photons: revealing quantum fundamentals

**DOI:** 10.1098/rsta.2023.0327

**Published:** 2024-12-24

**Authors:** Miles Padgett

**Affiliations:** ^1^ School of Physics and Astronomy, The University of Glasgow, Glasgow, G12 8QQ, UK

**Keywords:** orbital angular momentum, spatial modes, single photons

## Abstract

In 1992, Allen *et al*. (Allen L, Beijersbergen MW, Spreeuw RJC, Woerdman JP. 1992 Orbital angular momentum of light and the transformation of Laguerre-Gaussian laser modes. *Phys. Rev. A* **45**, 8185–8189. (doi:10.1103/physreva.45.8185)) published their seminal paper on the orbital angular momentum of light, drawing together seemingly unrelated themes and ideas in optics. This breakthrough initiated a new area of optical science concerning the physics and applications of structured light beams. This orbital angular momentum is an important concept for both classical and quantum science, especially where the framing in terms of angular momentum demystifies some of the quantum properties of light. Loudon’s own work (Loudon R. 2003 Theory of the forces exerted by Laguerre-Gaussian light beams on dielectrics. *Phys. Rev. A* **68**, 013806. (doi:10.1103/PhysRevA.68.013806)) in this area focused on the interactions between light and matter where the orbital angular momentum extended his studies from linear impulses to rotational torques.

This article is part of the theme issue ‘The quantum theory of light’.

The 1992 paper *Orbital Angular-Momentum of Light and the Transformation of Laguerre–Gaussian Laser Modes* by Allen *et al*. [1] is a seminal paper that drew together seemingly disconnected research findings to create a new paradigm of understanding of optical momentum. The possibility of laser modes with a helical phase structure in the transverse plane had been recognized by Vaughan and Willetts [2] and refined by Harris *et al*. [3], their associated azimuthal energy flow recognized by Coullet *et al*. [4] and the means of creating them by the transformation of a Gaussian beam using forked diffraction gratings by Soskin *et al*. [5]. The work of Allen *et al*. also had parallels with earlier work by Nye & Berry on the presence of phase singularities (dislocations) in both acoustic and optical fields [6]. However, none of these earlier works made the link between helical phase fronts, their associated azimuthal component of the Poynting vector, their azimuthal energy flow and, hence, the inherent angular momentum that resulted. The insight of Allen and co-workers [1,7] was to deduce that a laser beam with helical phase, ϕ(θ), described in cylindrical coordinates by $\phi \left(\theta \right)=\exp\left(-l\theta \right)$ gave rise to an angular momentum equivalent to lℏ per photon. To distinguish this angular momentum from spin angular momentum arising from circular polarization, the angular momentum associated with these helical phase fronts was called ‘orbital angular momentum’. Beyond visible light, this orbital angular momentum has been generated in many other domains too, ranging from radio waves [8] and X-rays [9] to acoustics [10], electrons [11,12] and neutron beams [13]. Beyond the paraxial limit, the formal separation between spin and orbital angular momentum is subtle but remains valid [14].

Despite being quantified as a per photon value, much of the early work on the orbital angular momentum of light focused on its description in terms of fields rather than photons, the per photon value being deduced from the angular momentum to energy ratio of the light beam. This ratio approach had parallels with the original determination by Poynting of the spin angular momentum of light arising from circular polarization where the angular momentum to energy ratio of an electromagnetic was shown to be 1/*ω* [15]. However, even in the early years, this field-based interpretation of orbital angular momentum was not the whole story since it had already been recognized that the quanta of light (photons) must be able to carry an angular momentum in addition to its spin state. In the 1930s, Darwin reasoned that high-order transitions required an angular momentum exchange greater than ℏ [16]. In that work, the additional angular momentum was articulated to arise from the linear momentum acting about a radius vector. In essence, an additional ℏ of angular momentum is generated by the linear momentum of ℏ*k* acting about a radius of 1/*k*.

In modern times, the conceptual link between linear and angular momentum has been exemplified by considering the generation of orbital angular momentum through the use of a spiral phase plate [17]. A spiral phase plate is a transparent disc of a thickness that increases as a function of the azimuthal angle such that an incident plane wave is converted into a transmitted wave with helical phase fronts. A simple consideration of Snell’s Law, see figure 1, for the refraction of a transmitted light ray with a linear momentum equivalent to ℏ*k* per photon gives rise to an azimuthal component to the linear momentum of lℏ/*r* per photon and, hence, an angular momentum of lℏ [18]. As we see later, the use of this ray optic model and the various components of the linear momentum is surprisingly powerful as applied to modal capacity, nonlinear phase matching considerations and even the optical forces acting on dielectrics. From a quantum perspective, the uncertainty relation between angle and angular momentum [19] is consistent with the notion that a precise value of the orbital angular momentum requires there to be no angular restriction, i.e. that the intensity distribution of the beam is circularly symmetric.

**Figure 1.**
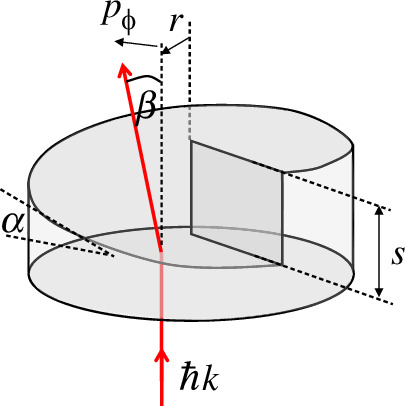
The ray optical analysis of a spiral phase plate of refractive index, n, of step height of s=ℓλ/(n−1) and a local surface gradient of α=s/2πr leading to a linear momentum with an azimuthal component of $p_{\emptyset }=\hbar k\left(n-1\right)s/2\pi r$ and, hence, an orbital angular momentum per photon of lℏ.

Much more recently than the work of Darwin, the interaction of orbital angular momentum carrying Laguerre–Gaussian laser beams with atoms was considered in the mid-1990s by Babiker and co-workers [20–22], but most of that work solely analysed the interaction of the laser beams with the centre of mass motion of the atoms and/or their associated recoil/Doppler shift. The observation of the transfer of the orbital angular momentum to an atomic state came a further 20 years later when Schmiegelow *et al*. held a single trapped ion exactly on the beam axis of a Laguerre–Gaussian mode and excited a high-order transition with a combination of spin and orbital angular momentum [23].

Immediately post Allen *et al*. [1], perhaps the most captivating line of work in orbital angular momentum was the observation of the transfer of orbital angular momentum to small particles which were set into rotation first about their own axis [24,25] and subsequently about the beam axis [26]. Another line of work in the mid-1990s was investigating the role that orbital angular momentum might play in nonlinear optics. Initially, this work considered only the frequency-doubling process within a nonlinear crystal [27], where the mathematical squaring of the field resulted in a doubling of both the frequency and the orbital angular momentum of the beam [28]. Unsurprisingly, this doubling corresponded to a conservation of both energy and momentum within the optical fields. This conservation of momentum of orbital angular momentum within the optical field persists beyond second-order processes to, for example, high-harmonic generation too [29].

However, frequency up-conversion is not the only possible process within a nonlinear crystal, and in 2000, Arnaut & Barbosa [30] considered the conversation of orbital angular momentum in the down-conversion process, a process where a high-frequency pump photon is absorbed, and two lower-frequency signal and idler photons are created. In this down-conversion, the orbital angular momentum of the signals and idler photons, although individually only loosely constrained, must conserve angular momentum and, therefore, add up to that of the pump photon.

In 2001, Zeilinger and co-workers combined this insight on the conservation of orbital angular momentum within second-order nonlinear optics [28,30] with the generation and measurement of orbital angular momentum using forked diffraction gratings [5] and with their own extensive knowledge of experiments to demonstrate quantum correlations and entanglement [31]. In their seminal paper, they were able to demonstrate the quantum entanglement between the orbital angular momentum states of the down-converted photons [32]. The key to this work was showing not only that the orbital angular momentum was conserved when measuring integer values of l but also that the correlations remain strong for arbitrary superpositions of orbital angular momentum states and that the strength of the correlations depends upon both the magnitude and argument of these superpositions. These superpositions were created by deliberately displacing the forked diffraction grating away from the beam axis, where the size of the displacement sets the magnitude of the states, and the direction of the displacement sets their relative phase [33]. Beyond orbital angular momentum itself, this ground-breaking work of Zeilinger and co-workers opened the field of high-dimensional quantum entanglement, highlighting how photons were not restricted to the two-dimensional Hilbert space of polarization and spin angular momentum.

Building on the work of Zeilinger and co-workers, rather than displacing the hologram, by considering analogous experiments in two-dimensional Hilbert spaces, the Poincaré sphere (for polarization) and the Bloch sphere more generally allows easy analogies between spin (i.e. polarization) and orbital angular momentum (i.e. modes) to be made [34] (see figure 2). Subsequent work used measurements made on the rotated Hermite–Gaussian modes to show a violation of Bell-type inequalities in the same fashion as for polarization [35]. These same analogies were later exploited to obtain the corresponding density matrices, allowing quantum tomography of the entangled state [36].

**Figure 2.**
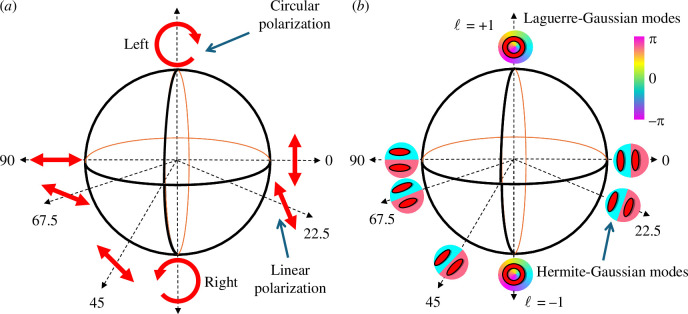
(*a*) The Poincaré sphere for polarization and (*b*) an equivalent Block sphere for the l±2 subspace showing the equivalent measurements required to show a violation of the Bell inequality for orbital angular momentum [35].

Beyond the two-dimensional state space, the ability to observe the high-dimensionality of the quantum state was not a completely new concept, for example the original Einstein–Podolsky–Rosen paradox [37] relating to correlations between the positions or momenta of separated photons (particles) is clearly also of high dimensions (i.e. many different possible positions and many different possible momenta). What is less clear in the position–momentum formulation is access to the range of other mutually unbiased measurement bases or the measurement of complex superpositions of states in any basis. By contrast, orbital angular momentum creates a well-defined basis of high dimensionality where other unbiased basis can be expressed as modal superpositions of the orthogonal orbital angular momentum states [38]. One such unbiased basis is an angular position which is the Fourier transform of the orbital angular momentum states [39] and, therefore, lends itself to establishing the angular equivalent of the Einstein–Podolsky–Rosen paradox [40] or even the high-dimensional equivalent to the Bell inequality [41]. This entanglement of the orbital angular momentum within a high-dimensional state space can be additional to the entanglement of other variables, one example being the simultaneous, or hyper-, entanglement of many variables at the same time, including both orbital and spin angular momentum [42].

Although the dimensionality of the orbital angular momentum state-space is, in principle, unbounded, the dimensionality of any experimental realization is bounded by the precise experimental configuration. At a fundamental level, the dimensionality of a state-space based on spatial modes is independent of the modal basis used to describe it; the dimensionality is instead set by some parameter of the optical system used to prepare or measure the states. For example, if the optical system is based upon single-mode optical fibre, then the only mode which can propagate is the fundamental Gaussian mode and, hence, the dimensionality of the spatial-mode state-space is one. More generally, the modal capacity of multi-modal optical systems is often characterized in terms of their etendue, i.e. the product of the system aperture and its numerical aperture, but there are many similar concepts ranging from the characterization of the modal output of a laser in term of its *m*-squared or the number of modes supported by a cavity in terms of the Fresnel number. For an imaging system, the number of modes supported is simply that of the number of resolvable pixels within the field of view. Irrespective of the modal capacity of the optical system, within parametric down-conversion, there is a restriction on the number of orthogonal spatial modes produced, typically several thousand [43]. This restriction is set by the phase-matching process in the nonlinear crystal that sets an upper limit to the numerical aperture of the down-converted light, which, when combined by the diameter of the pump beam, defines the limiting etendue and the orbital angular momentum bandwidth [44]. For the specific case of orbital angular momentum, entanglement has been observed between modes corresponding to several hundred ℏ per photon [45], reflecting the large modal capacity of the system. The range of orbital angular momentum values of the down-converted light is maximized by maximizing the number of down-converted modes, which implies both large-diameter pump beams and thin nonlinear crystals. This bandwidth can then be further increased by careful tuning of the phase-matching condition to increase the numerical aperture of the down-converted light [44].

Beyond being a demonstration of high-dimensional quantum mechanics, a question arises as to how this high-dimensional entanglement might be applied to quantum processing and/or quantum communications. Beyond the suggestions made within the 2001 paper by Zeilinger *et al*. [32], an early realization of the potential of high-dimensional entanglement orbital angular momentum states was made by Torner *et al*. [46] who considered how photons could be both prepared and measured in complex superposition of states within a high-dimensional Hilbert space.

The first, in principle, demonstration of using orbital angular momentum as the main degree of freedom in a practical communication system was in 2004 [47], but many other demonstrations have followed, some reaching extremely high data rates [48] or long-range, free-space [49] or fibre-based [50,51] operation. The use of orbital angular momentum has also been shown within a quantum key distribution protocol where the high dimensionality of the state space has led to an increase in the data transmitted per detected photon [52]. For a recent overview of quantum cryptography using structured light, see [53].

From the more general standpoint of quantum information processing, as mentioned above, compared to polarization (i.e. spin angular momentum), the orbital angular momentum of lights offers both an unbounded increase in the dimensionality of the state-space and measurable unbiased bases [38]. One example of using this additional degree of freedom has been the refinement of quantum teleportation [54]. Although quantum teleportation has perhaps little in common with teleportation in the science fiction sense of the phrase, it is intriguing never-the-less. In principle, measuring a single variable and copying it to another system is straightforward, but copying multiple and complementary variables (e.g. position and momentum) from one system to another is prevented by the no-cloning theorem [55]. However, through teleportation, the full quantum state can be copied from one particle to another using entangled photons, without violating the no-cloning theorem since the process fundamentally destroys the original state of the particle through measurement. These quantum teleportation protocols were originally based on polarization but have now been extended to multiple degrees of freedom and higher dimensional states including orbital angular momentum [56]. The high dimensionality of the orbital angular momentum state also lends itself to experiments to explore the entanglement between larger numbers of particles [57,58], a stepping stone to many protocols in quantum communication, networks and processing.

Looking to the future, perhaps the legacy of orbital angular momentum is not the momentum property itself but rather that the spatial structuring of light beams both in their intensity and phase is important both for exploring fundamental physics and driving forward applications. From an experimental point of view, much of this previous and ongoing work is made possible through the availability of spatial-light modulators, giving computer control of the spatial structure of the beam. For an extensive review of future directions, see [59].

Loudon, too, was active in the understanding of both the classical and quantum properties of orbital angular momentum. His work built upon his extensive studies in the interaction forces between light and dielectric materials and how this scales with the refractive index, relating to the Abraham–Minkowski dilemma [60,61]. In 2003, Loudon extended his work on these interactions from the consideration of the linear momentum to the angular momentum and Laguerre–Gaussian mode specifically [62]. His principal finding was that an analysis in terms of the Lorentz force predicts a torque corresponding to the ‘Einstein box’, i.e. Abraham interpretation, scaling inversely with the square of the refractive index and the same answer as one would expect as from a ray optics argument (see figure 3). The Einstein box argument equates the azimuthal displacement of the energy associated with the transmitted optical ray with the counter rotation of the mass–energy equivalent of the dielectric disc [63]. Although the prediction of the Abraham interpretation is clear, convincing experimental confirmation of these predictions remains an open and intriguing question.

**Figure 3.**
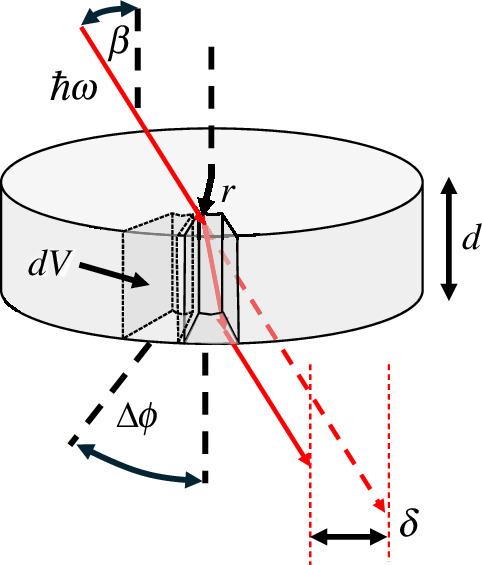
The Einstein box argument applied to the rotation of a dielectric disc. A ray representative of a beam carrying orbital angular momentum with a skew angle, β, with respect to the optical axis, incident on a transparent disc of thickness *d*, is subject to an azimuthal displacement, δ, of the energy, ℏω, which is equated to the counter rotation, Δϕ, of the mass energy equivalent, $mc^{2}$, of the volume element dV.

This last work was my own opportunity to work directly with Rodney and what a pleasure it was. I remember, as a mid-career researcher, plucking up the courage to contact Professor Loudon directly to ask for some physics advice. Having arranged to call, I was answered by a cheery voice that told me how much he was looking forward to the discussion but that he was unlikely to be able to explain anything that I had not already understood since my existing work had shown that I was myself more expert in the topic than he. In reality, and as the subsequent discussion confirmed, I most certainly was not more expert, but it is a hallmark of the man that his first thought was to put his junior colleague, me, at ease. Rodney was an exceptional scholar and, more importantly, everything good about being a gentleman, a walking example of how to behave to us all.

## Data Availability

This article has no additional data.
